# NHS Health Check attendance is associated with reduced multiorgan disease risk: a matched cohort study in the UK Biobank

**DOI:** 10.1186/s12916-023-03187-w

**Published:** 2024-01-23

**Authors:** Celeste McCracken, Zahra Raisi-Estabragh, Liliana Szabo, John Robson, Betty Raman, Anya Topiwala, Adriana Roca-Fernández, Masud Husain, Steffen E. Petersen, Stefan Neubauer, Thomas E. Nichols

**Affiliations:** 1 Division of Cardiovascular Medicine, Radcliffe Department of Medicine, University of Oxford, National Institute for Health Research Oxford Biomedical Research Centre, Oxford University Hospitals NHS Foundation Trust, Oxford, OX3 9DU UK; 2 William Harvey Research Institute, NIHR Barts Biomedical Research Centre, Queen Mary University of London, Charterhouse Square, London, UK; 3 Barts Heart Centre, St Bartholomew’s Hospital, Barts Health NHS Trust, West Smithfield, London, UK; 4 Semmelweis University, Heart and Vascular Center, Budapest, Hungary; 5 Wolfson Institute of Population Health, Queen Mary University of London, London, UK; 6 Big Data Institute, Li Ka Shing Centre for Health Information and Discovery, Nuffield Department of Population Health, University of Oxford, Oxford, OX3 7LF UK; 7 Perspectum Ltd., Gemini One, Oxford, UK; 8 Nuffield Department of Clinical Neuroscience, University of Oxford, Oxford, UK; 9 Division of Clinical Neurology, John Radcliffe Hospital, Oxford University Hospitals Trust, Oxford, UK; 10 Department of Experimental Psychology, University of Oxford, Oxford, UK; 11 Wellcome Centre for Integrative Neuroimaging (WIN FMRIB), University of Oxford, Oxford, UK; 12 Health Data Research UK, London, UK; 13 Alan Turing Institute, London, UK

**Keywords:** NHS Health Check, Primary care, Preventive medicine, General medicine, Public health

## Abstract

**Background:**

The NHS Health Check is a preventive programme in the UK designed to screen for cardiovascular risk and to aid in primary disease prevention. Despite its widespread implementation, the effectiveness of the NHS Health Check for longer-term disease prevention is unclear. In this study, we measured the rate of new diagnoses in UK Biobank participants who underwent the NHS Health Check compared with those who did not.

**Methods:**

Within the UK Biobank prospective study, 48,602 NHS Health Check recipients were identified from linked primary care records. These participants were then covariate-matched on an extensive range of socio-demographic, lifestyle, and medical factors with 48,602 participants without record of the check. Follow-up diagnoses were ascertained from health records over an average of 9 years (SD 2 years) including hypertension, diabetes, hypercholesterolaemia, stroke, dementia, myocardial infarction, atrial fibrillation, heart failure, fatty liver disease, alcoholic liver disease, liver cirrhosis, liver failure, acute kidney injury, chronic kidney disease (stage 3 +), cardiovascular mortality, and all-cause mortality. Time-varying survival modelling was used to compare adjusted outcome rates between the groups.

**Results:**

In the immediate 2 years after the NHS Health Check, higher diagnosis rates were observed for hypertension, high cholesterol, and chronic kidney disease among health check recipients compared to their matched counterparts. However, in the longer term, NHS Health Check recipients had significantly lower risk across all multiorgan disease outcomes and reduced rates of cardiovascular and all-cause mortality.

**Conclusions:**

The NHS Health Check is linked to reduced incidence of disease across multiple organ systems, which may be attributed to risk modification through earlier detection and treatment of key risk factors such as hypertension and high cholesterol. This work adds important evidence to the growing body of research supporting the effectiveness of preventative interventions in reducing longer-term multimorbidity.

**Supplementary Information:**

The online version contains supplementary material available at 10.1186/s12916-023-03187-w.

## Background

With ageing populations globally, the increasing multimorbidity burden is having a serious impact on healthcare costs, disability, and mortality [1]. Although multimorbidity prevention is now recognised as a high priority [2], and forms part of the National Health Service (NHS) Long Term Plan [3], there are few established strategies with known effectiveness. Disease prevention is particularly challenging when conditions develop over a long period of time without symptoms, meaning that disease is often only detected once it has progressed to advanced stages. Therefore, when it comes to multimorbidity prevention, a proactive—rather than reactive—approach may be required [4].

The NHS Health Check programme is a preventative primary care intervention launched in England, UK, in 2009, designed to identify individuals at risk for heart disease, stroke, diabetes, and kidney disease [5, 6]. Briefly, under the NHS Health Check protocol, healthy adults aged 40–74 are invited to visit their primary care team for basic physical checks, blood tests, and health behaviour questions, after which attendees are offered support and services to help prevent or delay the onset of these conditions. With a similar and slightly expanded protocol, the Scottish version (“Keep Well”) was launched in 2006 and is specifically targeted towards areas of socio-economic deprivation [7, 8].

There is a growing body of evidence around the administration and uptake of the NHS Health Check [9, 10]. However, there is much less research on the associations between NHS Health Check and subsequent health outcomes and the effectiveness of the programme in disease prevention. Although there is some existing work documenting the short-term effects of NHS Health Check [11, 12], evidence regarding longer-term outcomes is proving to be particularly challenging to find. Additionally, the evidence we have often fails to account for follow-up outcomes across multiple disease endpoints. Preventive public health programmes are also in place in other nations [13, 14]; however, similar gaps in evidence exist in relation to the value of such strategies in promoting long-term health.

In this study, we draw upon the UK Biobank resource to identify participants who have received an NHS Health Check and to observe any new diagnoses received during the follow-up period. The objective of this study is to compare the health outcomes across multiple morbidities, in participants with and without evidence of receiving an NHS Health Check.

## Methods

This is an observational, case–control study reported in accordance with the STROBE guidelines (see Additional file 1: Checklist S1).

### Study sample

**Fig. 1:**
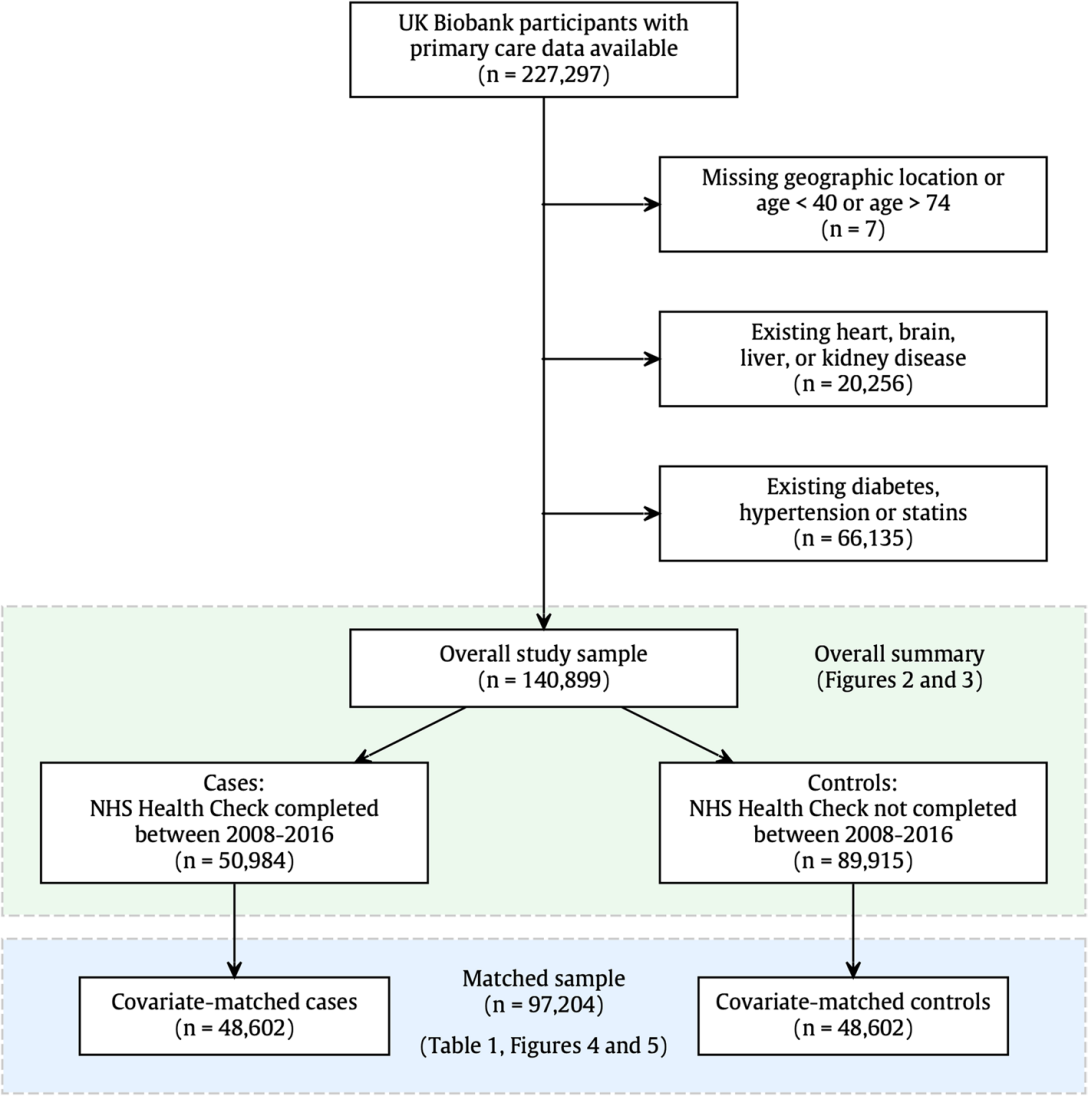
Study sample selection. Inclusion for this study was dependent on participants having primary care data available and eligibility for the NHS Health check. In other words, participants were between 40 and 74 years and had no existing diagnoses that would normally mean regular primary care monitoring. Overall summaries in Figs. 2 and 3 relate to the full study sample (*n* = 140,899), and outcome modelling was performed with the covariate-matched sample (*n* = 97,204), with details shown in Table 1 and Figs. 4 and 5

The UK Biobank is a UK-wide prospective cohort study, with baseline recruitment taking place between March 2006 and October 2010. At the time of this study, there were 227,297 participants who had completed consent for their primary care records to be made available and who had confirmed primary care records in the database (Fig. 1). From this pool of participants, 7 participants had missing geographical location or were outside the age range for the NHS Health Check. A further 20,256 participants (9%) were excluded as ineligible for an NHS Health Check due to an existing diagnosis of heart, brain, liver, or kidney disease, and 66,135 participants (31%) were excluded due to statins prescription or an existing diagnosis of diabetes or hypertension. The NHS Health Check criteria specifically exclude people with these conditions as they are considered to be already identified and managed by their NHS providers. Thus, 140,899 participants were used as the study sample.

### Case–control matching

In order to reduce confounding from known sources, we applied an extensive case–control matching algorithm, to produce a control group that was matched one-to-one with NHS Health Check recipients using nearest neighbour propensity score matching with a calliper width of 0.2, as per published recommendations [15]. Matching was conducted with respect to demographic features (geographical region, age, sex, ethnicity, Townsend deprivation index, education), family history of disease (stroke, heart disease, dementia), physical measures (body mass index, waist-to-hip ratio, systolic blood pressure at baseline), and health behaviours (smoking, alcohol intake frequency, physical activity, and daily vegetable intake). More than 95% of cases (NHS Health Check recipients) received a high-quality match, and non-matching participants were dropped from the analysis. A review of the excluded participants indicated that although they tended to be younger, more deprived, and more obese, the main reason for matching failure was the requirement for one-to-one matching within a geographical region. In other words, the matched control for a London health check recipient must also be from London. From this process, we derived a matched sample of 97,204 participants, with 48,602 participants in each exposure group (Fig. 1).

### The intervention: NHS Health Check

**Fig. 2:**
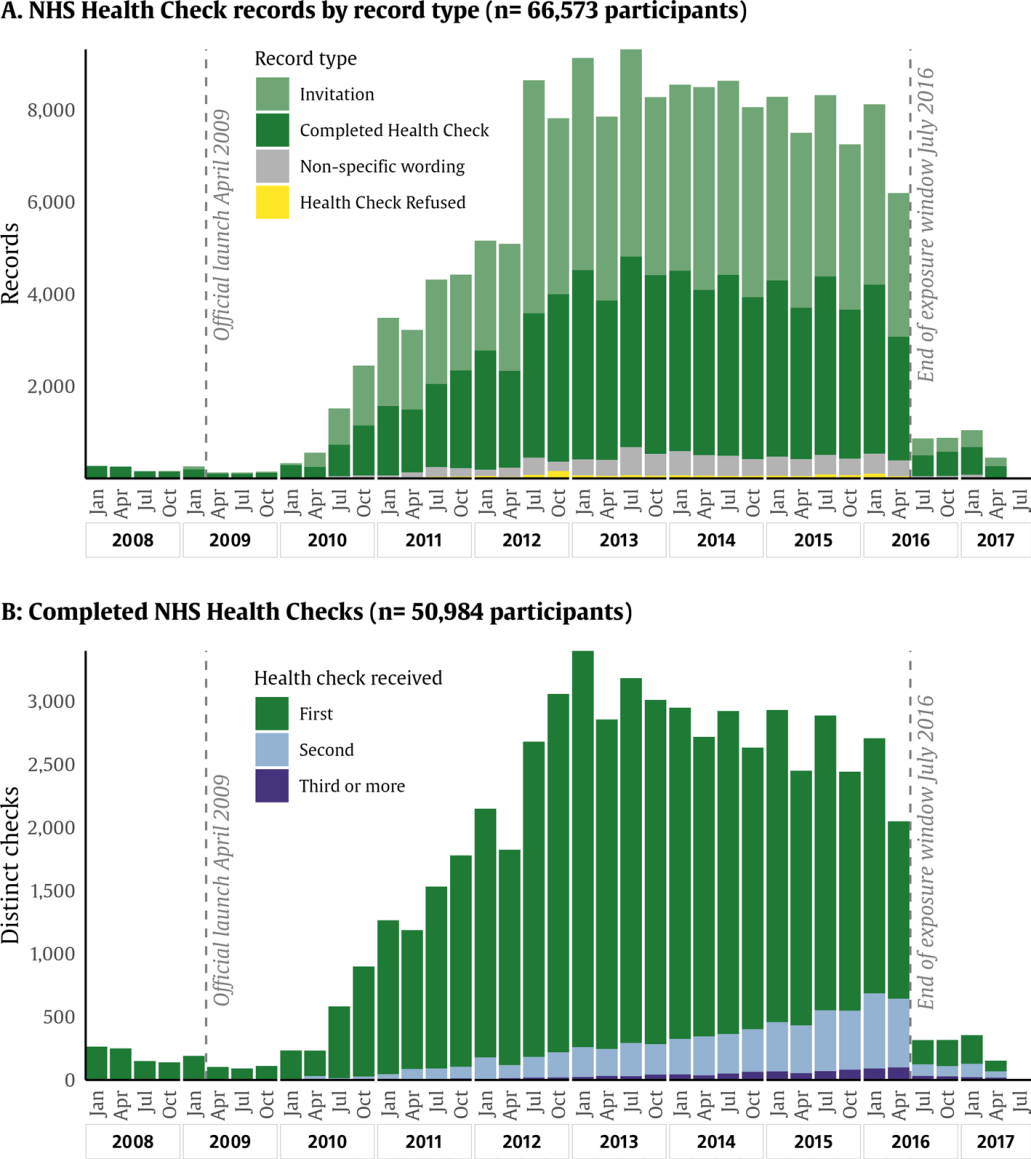
NHS Health Check records by year and record type. Distributions describing NHS Health Check records in the UK Biobank. **A** The distribution of records tagged with NHS Health Check identifiers, coloured by record type, whether the record describes a completed health check, an invitation, a refusal, or a descriptive tag (not necessarily implying completion or refusal). **B** The distribution of completed health checks, with blue showing the first recorded NHS Health Check and with green and yellow showing follow-up health checks. Participants are included from January 2008 to July 2016

To identify participants who had received an NHS Health Check (or its Scottish equivalent), we performed a text search of primary care clinical records, for the following phrases “keep well, health check, check-up, healthy start, healthy lifestyle, diabetes prevent, well man, well woman, well adult”. This search returned 283,536 records between April 2006 and December 2022. From here, we reviewed the record text to exclude other types of health checks (healthy lifestyle, diabetes management, carer health checks, and unspecified check-ups) and records that did not indicate NHS Health Check completion, for example, invitations (terms including invitation, email, letter), neutral descriptors (not appropriate, indicated, status), and health check refusal (declined, refused, did not attend). The remaining codes that were used to confirm NHS Health Checks are provided in Additional file 1: Table S1. Although the official nationwide launch took place in April 2009, there were some areas that participated in a pilot programme in the year leading up to this date [16]. Within the UK Biobank resource, coverage for primary care records was high up to mid-2016, when the availability of records dropped sharply. Therefore, the exposure window was set between 1 January 2008 and 30 June 2016. The count of records for health check exposure by year is illustrated in Fig. 2. For modelling purposes, we considered the date of exposure to be the date of the first completed NHS Health Check.

### Ascertainment of outcomes

Existing disease at baseline was assessed via both self-report and linked records (hospital and primary care). Incident disease after baseline was ascertained via primary care linked records, hospital records, and death registry (Additional file 1: Table S2), using published code lists where available [17–19]. We included the following outcomes: hypertension, diabetes, hyperlipidaemia, stroke (any type), all-cause dementia, myocardial infarction, atrial fibrillation, heart failure, acute kidney injury, chronic kidney disease (stage 3, 4, or 5), fatty liver disease, alcoholic liver disease, liver cirrhosis, liver failure, cardiovascular mortality (death with a primary cause between ICD I00–I80), and all-cause mortality. Participants were followed up until the end of October 2022. All patient consent procedures were completed by the UK Biobank, and details of record linkage in the UK Biobank have been published previously [20].

### Definition of covariates

Age, sex, and all other covariates were recorded at baseline. Modelling covariates were selected based on their known associations with outcome risk in previous research; these include age, sex, geographical region, Townsend deprivation score, ethnicity (White/non-White), post-secondary education (yes/no), body mass index, waist-to-hip ratio, current smoking (yes/no), systolic blood pressure, Charlson Comorbidity Index, alcohol intake frequency, physical activity, and fresh vegetable intake. Charlson Comorbidity Index was coded from existing conditions at baseline [21]. Physical activity was coded in summed metabolic equivalent task-minutes per week, based on the aggregation of physical activity fields according to published guidance [22]. To partition participants into geographical regions, we identified the coordinates of six major cities in the mainland UK (London, Bristol, Birmingham, Manchester, Newcastle upon Tyne, and Edinburgh) and then allocated each participant to their nearest city based on the rounded east and north coordinate provided at baseline. Some of the covariates had a small amount of missing values (less than 2%) that were imputed together using multiple imputation with chained equations [23] (summarised in Additional file 1: Table S3).

### Statistical analysis

We used graphical illustrations and simple descriptive statistics to outline the features of UK Biobank participants with primary care data and the uptake of the NHS Health Check. To assess the covariate-adjusted differences in outcomes between health check recipients and non-recipients, we used two main modelling approaches.

#### Time-varying Cox regression

We applied Cox survival modelling with the intervention (NHS Health Check date) coded in a time-varying fashion following the method outlined by Therneau and colleagues [24–26]. In this method, follow-up begins at baseline registration, and all participants begin the follow-up period with intervention-negative status, in other words, no NHS Health Check. Then, over time, some participants receive an NHS Health Check, so at that date, they acquire intervention-positive status. Survival models were adjusted by the full complement of covariates outlined above, as recorded at baseline. This analysis makes use of the most amount of data and allows time-based differences in exposure effect to be observed more easily but could still be biased due to residual confounding.

#### Aligned-start Cox regression

In this analysis, we aligned the intervention window between cases and controls in a manner similar to Sebuødegård and colleagues [27] and then applied proportional hazards regression in the conventional sense. Here, the follow-up period is defined to begin at the date of the completed NHS Health Check, with outcome times aligned accordingly. Each control participant (without an NHS Health Check) is followed up from the intervention date of his or her matched pair. Full follow-up lengths were compared and were not significantly different between the groups. In aligned-start analyses, ages were updated to be consistent with the intervention date, with the remainder of covariates as measured at baseline. This method provides better quality control for known confounding but involves the loss of data, and hence loss in power.

Survival models in both approaches above were adjusted by age, sex, geographical region, Townsend deprivation score, ethnicity, post-secondary education, body mass index, waist-to-hip ratio, current smoking, systolic blood pressure, Charlson Comorbidity Index, alcohol intake frequency, physical activity, and fresh vegetable intake. Aligned-start models were additionally adjusted by the length of time since registration. Multiple testing correction using a false discovery rate of 5% was applied to identify significant *p*-values across all models.

#### Further time adjustments

Prior research has identified that NHS Health Check receipt was associated with increased detection and diagnosis of prevalent (but unrecognised) diabetes, hypertension, and cardiovascular and kidney diseases [9]. Therefore, we conducted the survival modelling with three-time exclusion settings: (a) include all outcomes, (b) exclude outcome events in the first 12 months after NHS Health Check, and (c) exclude outcome events in the first 24 months after NHS Health Check. In epidemiological research, it is common to exclude events that occur in the first few years of follow-up when studying the relationship between an exposure and a disease outcome. This latency period intends to reduce bias from reverse causation, which occurs when the main biological processes creating the disease outcome precede the exposure. By removing events that occur in the early years of follow-up, we hope to observe associations between the exposure and disease outcome that are not due to pre-existing disease influencing exposure status (see Rothman and colleagues, p.218–219 [28]), and examples [29, 30]. We tested the proportional hazards assumption for the effect of NHS Health Check on the outcome risk using a chi-square test and graphical display of Schoenfeld residuals. In sensitivity analysis, we re-ran the aligned start Cox models with stratified time periods (Therneau and colleagues [24], Sect. 4). This approach has the lowest power of all, as each coefficient is calculated only using the outcomes within each time stratum.

#### Unmeasured confounding

Finally, we applied the *e*-value methodology described by VanderWeele and colleagues [31] to evaluate the potential nullifying impact of unobserved confounding variables. We calculated *e*-values and their lower bounds for each significant result and provided a translation of these into “years of ageing equivalent” to aid intuitive understanding of their relative strength.

## Results

### Description of NHS Health Check in the UK Biobank

Within the overall sample (*n* = 140,899), 66,573 participants (47%) received at least one invitation for an NHS Health Check between January 2008 and June 2016 (Fig. 2). Of those who were invited, 50,984 participants (77% of those invited, 36% of full sample) received an NHS Health Check. Within recipients, most received only one NHS Health Check (44,910, 88%), 10.5% received a second NHS Health Check, and 1.4% received three or more.

**Fig. 3:**
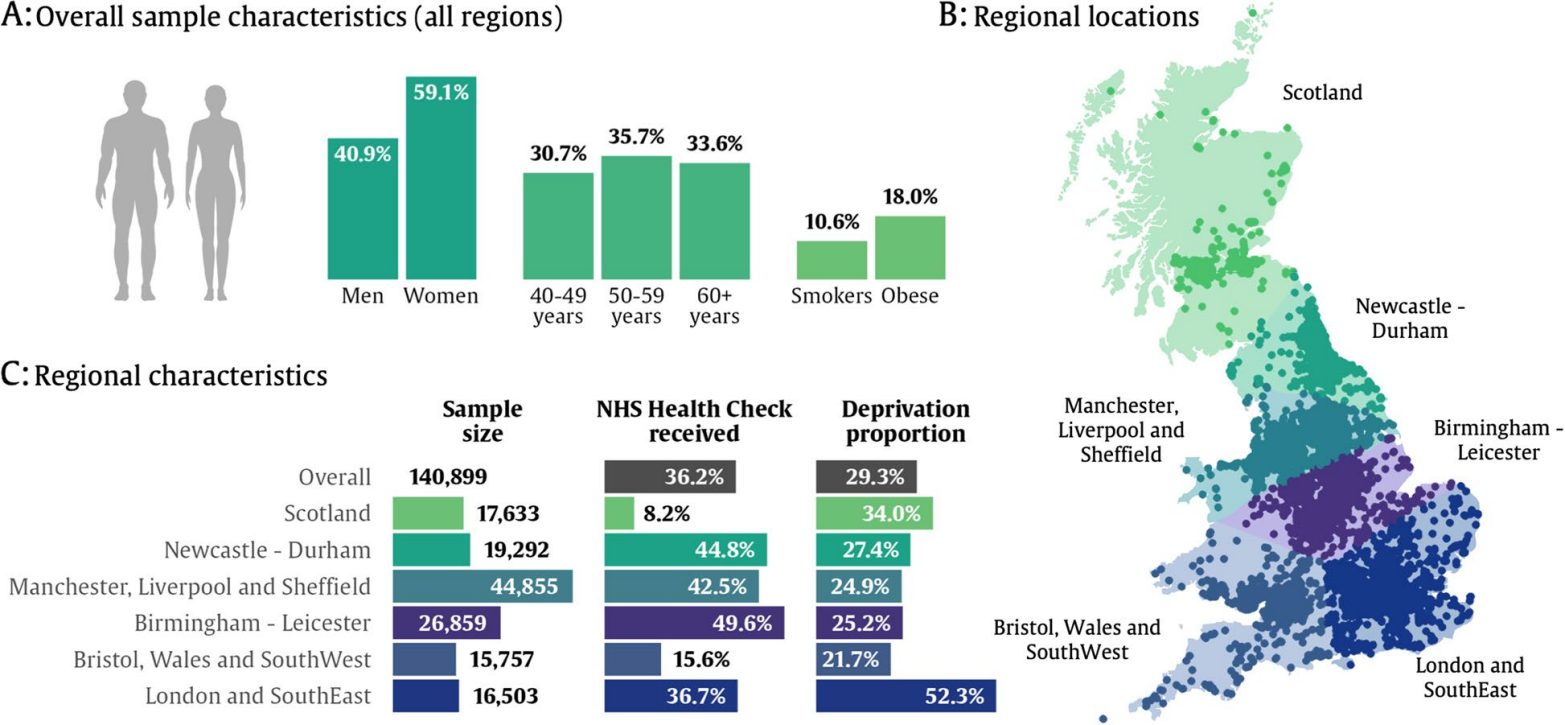
Summary of NHS Health Check in UK Biobank participants. **A** Overall summaries for the sample of UK Biobank participants with primary care available (*n* = 140,772). **B** Summary of geographical locations. **C** Sample characteristics by geographical region. Deprivation proportion refers to the proportion of the sample that fall above the UK median for socio-economic deprivation according to reported Townsend deprivation scores and quintiles from the 2011 census

In the full sample, there were more women than men (59% compared to 41%), with participants fairly evenly spread across the age range from 40 to 70 years at baseline (Fig. 3). Eighteen per cent of participants were obese (with body mass index > 30 kg/m^2^), and 10.6% were current smokers. Overall, 29.3% of participants had a Townsend deprivation index above the 2011 UK median indicating lower-than-average deprivation in the study sample [32]. This varied by geographical region, with London participants having the most nationally representative deprivation with 52.3% of participants above the UK median. NHS Health Check uptake rates also varied by geographical region, with the highest uptake in Birmingham, Manchester, and Newcastle regions (all above 40%), with the lowest uptake observed in Scotland (8.2%).

### The matched modelling set and observed outcomes

The matched subset used for modelling was very similar in profile to the overall study set, with 95% of NHS Health Check recipients (48,602/50,984) receiving a high-quality matched control pair (Table 1).

**Table 1:** Characteristics of NHS Health Check recipients and matched controls

Category	Variable	NHS Health Check recipients (*n* = 48,602)	Matched controls (*n* = 48,602)
Demographics	Age 40–49 years	13,536 (27.9%)	13,536 (27.9%)
	Age 50–59 years	17,998 (37.0%)	17,998 (37.0%)
	Age 60 + years	17,068 (35.1%)	17,068 (35.1%)
	Women	29,040 (59.8%)	29,040 (59.8%)
	Men	19,562 (40.2%)	19,562 (40.2%)
	Townsend deprivation index > UK median	13,950 (28.7%)	14,152 (29.1%)
	Non-White ethnicity	2048 (4.2%)	2046 (4.2%)
	Post-secondary education	29,986 (61.7%)	30,135 (62.0%)
Family history	Family history of stroke	12,664 (26.1%)	12,650 (26.0%)
	Family history of heart disease	20,280 (41.7%)	20,272 (41.7%)
	Family history of dementia	7226 (14.9%)	7229 (14.9%)
Health parameters	Obesity (body mass index ≥ 30 kg/m2)	7957 (16.4%)	8130 (16.7%)
	Waist-hip ratio ≥ 1	2073 (4.3%)	2082 (4.3%)
	SBP at baseline ≥ 140 mmHg	15,309 (31.5%)	15,889 (32.7%)
	Current smoker	4640 (9.5%)	4738 (9.7%)
	Daily alcohol intake	9376 (19.3%)	9325 (19.2%)
	Inactive (< 600 MET-minutes/week)	11,326 (23.3%)	11,307 (23.3%)
	Salad/raw vegetables daily (< 2 Tbsp)	21,134 (43.5%)	21,099 (43.4%)
Geographical region exposure	Birmingham—Leicester	11,564 (23.8%)	11,564 (23.8%)
	Bristol, Wales, and Southwest	2411 (5.0%)	2411 (5.0%)
	London and SouthEast	5979 (12.3%)	5979 (12.3%)
	Manchester, Liverpool, and Sheffield	18,790 (38.7%)	18,790 (38.7%)
	Newcastle—Durham	8427 (17.3%)	8427 (17.3%)
	Scotland	1431 (2.9%)	1431 (2.9%)
	NHS Health Check completed	48,602 (100.0%)	–
	Average follow-up time (years)	9.1 (± 1.9)	9.0 (± 2.0)
Measured outcomes			
Metabolic	Hypertension	5603 (11.5%)	5398 (11.1%)
	Diabetes	1144 (2.4%)	1187 (2.4%)
	High cholesterol	3690 (7.6%)	3487 (7.2%)
Brain	Stroke	708 (1.5%)	770 (1.6%)
	All-cause dementia	562 (1.2%)	694 (1.4%)
Heart	Myocardial infarction	913 (1.9%)	1052 (2.2%)
	Atrial fibrillation	1660 (3.4%)	1759 (3.6%)
	Heart failure	739 (1.5%)	796 (1.6%)
Kidney	Acute kidney injury	1924 (4.0%)	2572 (5.3%)
	Chronic kidney disease	973 (2.0%)	946 (1.9%)
Liver	Fatty liver disease	502 (1.0%)	492 (1.0%)
	Alcoholic liver disease	67 (0.1%)	93 (0.2%)
	Liver cirrhosis	109 (0.2%)	161 (0.3%)
	Liver failure	43 (0.1%)	60 (0.1%)
Mortality	Cardiovascular mortality	279 (0.6%)	360 (0.7%)
	All-cause mortality	1989 (4.1%)	2568 (5.3%)

*MET* metabolic equvalent task. Cardiovascular mortality refers to any death with a primary cardiovascular cause (ICD I00–I80)

**Fig. 4:**
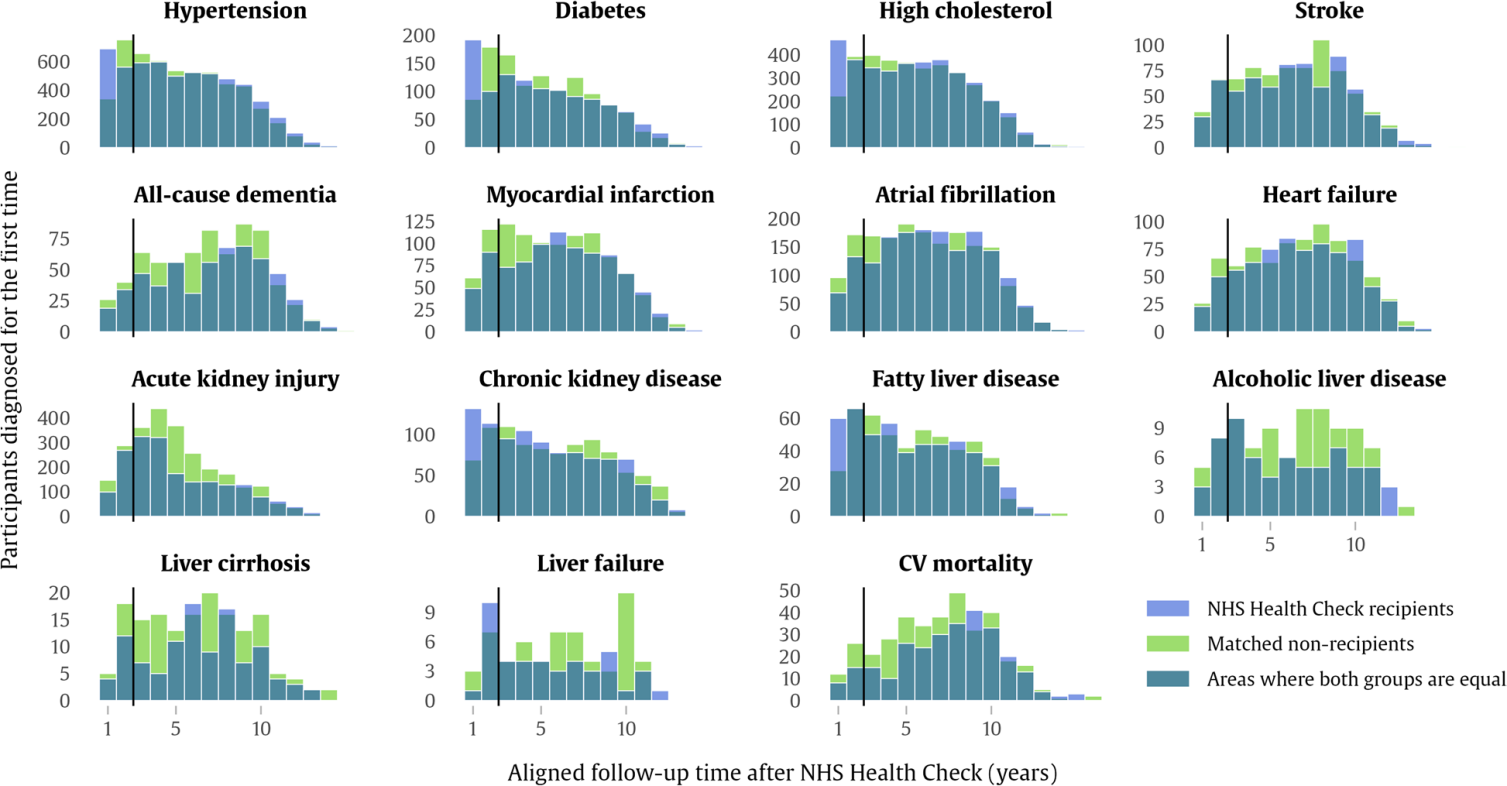
Outcomes during follow-up in NHS Health Check recipients and their matched controls. Bars show the counts of participants receiving condition diagnoses for the first time, in the years following the NHS Health Check in health check recipients and in the aligned exposure window in the covariate-matched control cohort. The dark teal shows the counts where both groups are equal, the bright purple indicates where the health check group counts are greater, and the bright green indicates where control counts are greater. The 2-year initial period following the health check is marked with a black vertical line

Characteristic of the UK Biobank more generally, the sample was mostly White ethnicity (4% other ethnicities), mostly physically active (23% inactive), with 19% of the sample consuming alcohol at least once per day. Table 1 also reports the outcome counts observed in the matched groups, along with their percentage rates. Many of the reported outcome rates are similar between the groups (hypertension, diabetes, stroke, heart failure, chronic kidney disease). For several outcomes, there are larger differences between cases and controls, for example, all-cause dementia (1.2% vs 1.4%), myocardial infarction (1.9% vs 2.2%), and acute kidney injury (4.0% vs 5.3%). In Fig. 4, histogram plots of outcome counts in NHS Health Check recipients are overlaid with the time-aligned outcome counts observed in their matched counterparts. Interestingly, for some outcomes (hypertension, diabetes, high cholesterol, chronic kidney disease, fatty liver disease and liver failure), more new diagnoses were recorded in NHS Health Check recipients in the initial 2-year period following the health check than in their matched pairs. On the other hand, following the 2-year mark, outcome counts were more likely to be equal or slightly higher in people who did not receive the NHS Health Check.

### NHS Health Check associations with outcomes

**Fig. 5:**
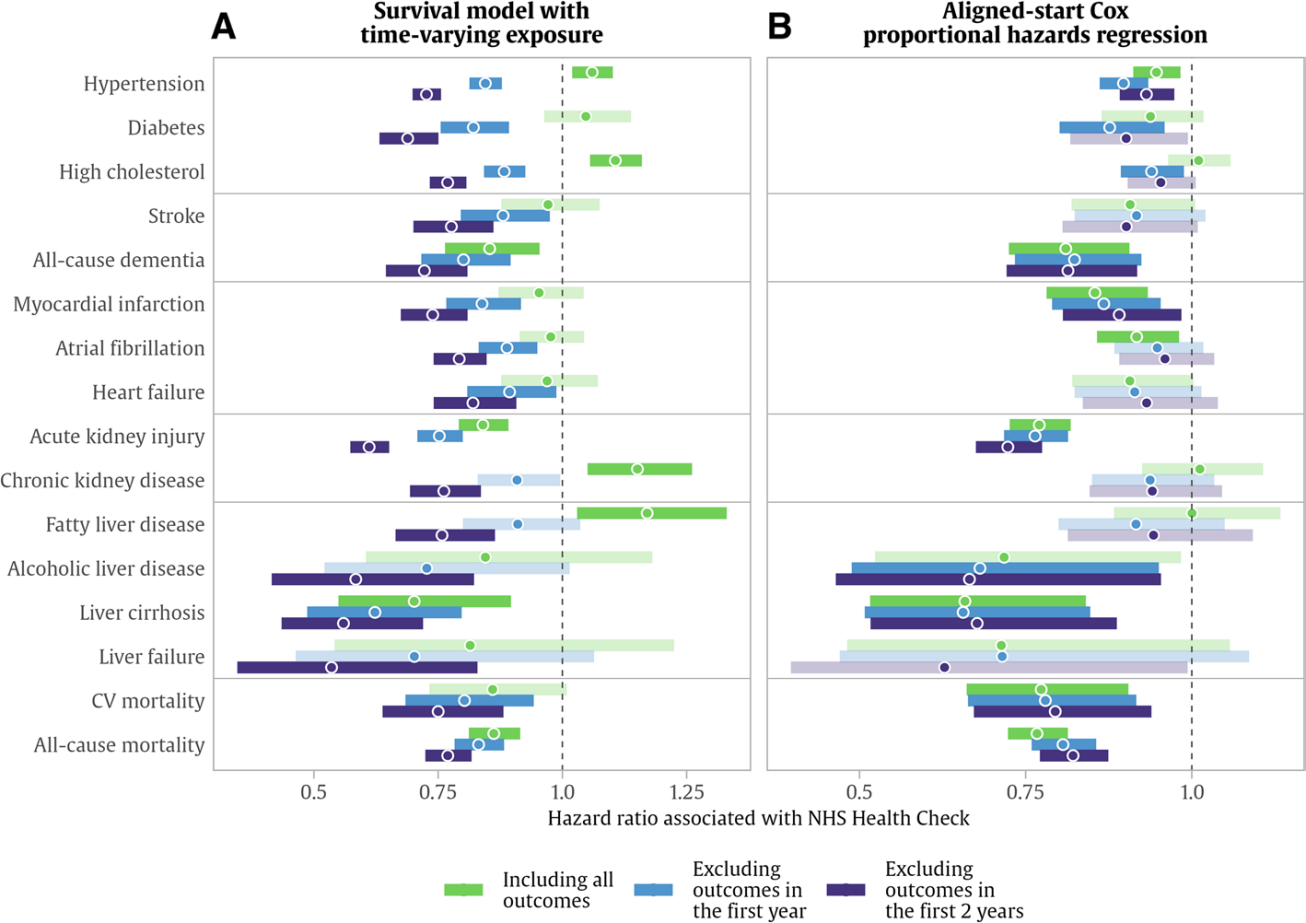
Associations between NHS Health Check and incident outcomes. Points represent hazard ratios, and horizontal bars represent 95% confidence intervals for hazard ratios from Cox proportional hazards models associating completed NHS Health Check with new diagnoses received during follow-up. **A** Hazard ratios from models with time-varying exposure. **B** Hazard ratios from models with aligned follow-up start times. The results shown in faded/greyed out colour were not significant after applying multiple testing correction with a 5% false discovery rate. Colours indicate that models were computed with three outcome exclusion settings (green (0) = including all outcomes, aqua blue (12) = excluding outcomes in the first 12 months after NHS Health Check, navy blue (24) = excluding outcomes in the first 24 months after NHS Health Check). Models are adjusted by age, sex, geographical region, Townsend deprivation score, ethnicity, body mass index, waist-hip ratio, smoking, systolic blood pressure, alcohol intake frequency, physical activity, daily vegetable/salad intake, and Charlson Comorbidity Index

Because the smoothed Schoenfeld residual curves and chi-square tests indicated departures from proportionality for several of the outcomes studied (Additional file 1: Fig. S1), we applied time stratification and two types of time-dependent modelling. In time-varying survival models, the time effect is demonstrated clearly. For some outcomes, the fully adjusted diagnosis rate was significantly higher in NHS Health Check recipients (Fig. 5 and Additional file 1: Table S4). When early events (in the first 12 months) were included, diagnoses of hypertension were 6% higher in the NHS Health Check group, with additional higher rates for high cholesterol (11%), chronic kidney disease (15%), and fatty liver disease (17%). However, when the initial 12 or 24 months were excluded from the model, the overall hazards across all outcomes were significantly lower in NHS Health Check recipients, with average risk reductions between 20 and 40% (hazard ratios between 0.6 and 0.8).

In aligned-start regression models, early-event time effects are much less pronounced (Fig. 5 and Additional file 1: Table S4). In these fully adjusted models, NHS Health Check recipients had significantly lower diagnosis rates for all-cause dementia (19% lower, hazard ratio (HR) 0.81), myocardial infarction (15% lower, HR 0.85), atrial fibrillation (9% lower, HR 0.91), acute kidney injury (23% lower, HR 0.77), liver cirrhosis (44% lower, HR 0.66), and all-cause mortality and cardiovascular mortality (both 23% lower, with HR 0.77).

The significant early effects observed in the main analysis were confirmed in sensitivity analysis by applying time stratification to the aligned-start models (Additional file 1: Fig. S2), with the addition of significantly higher rates of diabetes diagnosed in the first year of follow-up. Time-stratified models characterised significantly reduced longer-term risk for the same disease outcomes identified in the main aligned-start models.

Application of *e*-values to the regression results (Additional file 1: Table S5) found moderate robustness of most observed hazard ratios to unmeasured confounding. The results for acute kidney injury, liver cirrhosis, and myocardial infarction had stronger *e*-values (less likely to be rendered insignificant by unmeasured confounders), while the *e*-values for all-cause dementia and atrial fibrillation were weak, meaning that even quite a weak unmeasured confounding variable could render our result non-significant.

## Discussion

In this large community-based sample from the UK Biobank across an average of 9 years of prospective follow-up, receipt of the NHS Health Check was related to reduced long-term risk of all-cause and cardiovascular disease mortality and reduced incidence of disease across the liver, kidney, heart, and brain organ systems. In analyses including the early follow-up period, those who had the NHS Health check had increased detection of hypertension, high cholesterol, and chronic kidney disease. This effect was not observed in analyses restricting follow-up to beyond 24 months after the Health Check, indicating that these increased diagnoses indicate heightened detection of undiagnosed or quiescent pre-clinical disease, the treatment of which likely contributes to the reduction in health outcomes observed in the longer term.

The existing evidence on the effect of the NHS Health Check programme so far is mixed [9]. Some studies have found that the programme is associated with small reductions in the risk of cardiovascular disease and diabetes, while others have found no significant benefit. In a 5-year study of electronic health records from several London commissioning groups, Robson et al. found that NHS Health Check attendees had a higher short-term likelihood of a diagnosis of type 2 diabetes, hypertension, and chronic kidney disease and receive treatment with statins and antihypertensives [33]. These findings were subsequently confirmed in a large nationally representative sample (QDatabase) [34].

Using a similar approach to the current work, Chang and colleagues [35] used a matched sample from the Clinical Practice Research Datalink (CPRD, with *n* = 29,672 in each group) to find evidence of an overall reduction in cardiovascular risk as computed by QRISK2 in NHS Health Check participants, and a significant increase in diagnoses for hypertension and diabetes. This study was unable to detect differences in longer-term outcomes with a median follow-up time of 2 years.

Our findings corroborate these observations in a much larger independent cohort by demonstrating an increased diagnosis of incident hypertension, high cholesterol, and chronic kidney disease in the immediate period after the NHS Health Check. These conditions are typically asymptomatic with a long pre-clinical phase. As such, they are almost always identified through screening or opportunistic diagnosis. The longer follow-up duration in our study enabled us to extend existing knowledge by demonstrating that this apparent increase in diagnosis is confined to the first 2 years after the NHS Health Check, after which the risk of incident health events is significantly lower in recipients.

Our study is the first to demonstrate an association between receipt of the NHS Health Check and reduction in long-term risk of multiorgan outcomes. We do not assert that significant changes flow directly from a single 15-min appointment. It is more plausible that longer-term trajectory changes arise from (1) increased detection and treatment of risk factors (such as diabetes, hypertension, and high cholesterol), (2) more regular face-to-face reinforcement towards healthy behaviour change (like exercise and smoking cessation), and (3) better management of underlying active disease processes like atherosclerosis and atrial fibrillation. These processes work together to reduce the risk of long-term illnesses in NHS Health Check recipients. The initial health check visit sets up a relationship with the primary healthcare team, which may not otherwise occur in ostensibly healthy people. Although a recent review has found wide variation in practice [12], the health check system is specifically designed to facilitate access to further healthcare services, increasing subsequent healthcare utilisation and providing further opportunities for appropriate screening and risk factor optimisation.

The uptake of the NHS Health Check is an active area of research. Several recent studies [36, 37] have identified a range of factors influencing health check uptake. In 2018/2019, Public Health England published an uptake rate for England at 45.9% of those invited [9]. This figure is corroborated by a study of primary care records in England over a similar time period as our study, which reports an uptake rate of 52.6% of those invited [38]. The overall health check rate of 36.2% observed in this study is in line with an invitation rate of 80% of those eligible and an uptake rate of 45%.

Some studies have also found that NHS Health Check is associated with improvements in health behaviours such as smoking cessation, physical activity, and diet and with better management of hypertension and hypercholesterolaemia [11, 39]. But also, it has been found that the programme has not reached everyone who would benefit, particularly those in more deprived areas, and that the programme’s effectiveness may have been limited by a lack of follow-up and support for lifestyle changes [9]. Using a health economics modelling approach, Thomas and colleagues [40] did not report differences in the outcomes but still found that NHS Health Check would generate significant cost savings from improved detection of cardiovascular risk factors such as diabetes and high cholesterol.

## Limitations

We recognise that the UK Biobank cohort is known to be healthier on average than the general UK population [41, 42], meaning that the rates of incident disease observed in the current study are likely to be lower than the UK average incidence rates [43]. Similarly, while this study found significant differences in the outcome rates overall, the exact size of any effects would need to be validated in a nationally representative cohort.

In addition, self-selection bias is a very important factor to consider when assessing the effectiveness of any voluntary intervention like the NHS Health Check. People who engage with health screening programmes are systematically different in important ways from those who do not. They tend to have a more favourable social, lifestyle, and health profile compared to non-attenders. Studies evaluating the clinical value of screening programmes for the prevention of health outcomes are highly susceptible to confounding by these factors, which can be challenging to capture and include in modelling adjustments [34]. In our study, we created a comparator cohort of health check non-attenders matched to recipients on an extensive range of social, demographic, and clinical factors, with descriptive analyses confirming high-quality matching on variables considered. These findings suggest that the observed associations in our study may be inflated, but not fully explained, by self-selection bias in the NHS Health Check recipient group.

The UK Biobank is subject to participation bias [44]. The extensive measurements taken at baseline recruitment are likely to have made all participants more aware of their cardiovascular risk and in turn influenced their decision to independently take part in the NHS Health Check programme. For the purposes of this study, however, all participants (both cases and controls) would be subject to this bias equally.

Finding evidence linking any intervention with longer-term outcomes can be challenging, due to the differences in implementation times and the abundance of potentially confounding factors. Although we have adjusted for a number of important confounders, there could still be residual uncaptured confounding associated with UK Biobank membership and attendance at NHS Health Checks. While we have explored several regression methods, future work could study a larger matched cohort of participants receiving their first NHS Health Check at the same time, to remove potential confounding associated with time differences. Lastly, the intervention and outcome variables in this study are both defined via electronic health records; therefore, the accuracy of both measures is limited by the quality and timeliness of the data coding and the integrity of data linkage [45].

## Conclusions

This study demonstrates that proactive, well-designed preventive programmes such as the NHS Health Check can be effective in reducing longer-term disease outcomes, across multiple organ systems. Overall, our results suggest that the NHS Health Check has a role in preventing long-term diseases through risk factor modification and that this effect is independent of any risk-modifying behaviours the participants may pursue otherwise. While it remains challenging to gather evidence-based data to evaluate screening programmes, our work goes some way in addressing methodological issues that have hampered previous studies and provide further evidence in support of the NHS Health Check as a preventive public health programme. This research is an important addition to a growing body of work supporting the long-term benefits of a programme such as NHS Health Check and its effectiveness for potential multimorbidity prevention.

## Supplementary Information

**Additional file 1: Checklist S1.** STROBE Checklist. **Table S1.** UK Biobank-specific primary care codes used to identify NHS Health Check exposure. **Table S2.** UK Biobank fields and codes for ascertainment of outcomes. **Table S3.** Missing value imputation details for sample covariates. **Fig. S1.** Visualisation of the time-varying hazard effects. **Table S4.** Hazard ratios, 95% confidence intervals and p-values for associations between NHS Health Check and incident diagnoses. **Fig. S2.** Sensitivity analysis with stratified time periods. **Table S5.** E-value lower bounds for significant hazard ratios.

## Data Availability

This research was conducted using the UK Biobank resource under access application 59,867. The UK Biobank will make the data available to all bona fide researchers for all types of health-related research that is in the public interest, without preferential or exclusive access for any persons. All researchers will be subject to the same application process and approval criteria as specified by the UK Biobank. For more details on the access procedure, see the UK Biobank website: https://www.ukbiobank.ac.uk/enable-your-research/register.

## Abbreviations

UK: United Kingdom
NHS: National Health Service
ICD: International Classification of Diseases
HR: Hazard ratio
