# Supplementary material for: NHS Health Check attendance is associated with reduced multiorgan disease risk: a matched cohort study in the UK Biobank
Source: BMC Med. 2024 Jan 23;22:1. doi: 10.1186/s12916-023-03187-w (PMC10804500; doi:10.1186/s12916-023-03187-w)
Supplement: Supplementary file 1 — Additional file 1: Checklist S1. STROBE Checklist. Table S1. UK Biobank-specific primary care codes used to identify NHS Health Check exposure. Table S2. UK Biobank fields and codes for ascertainment of outcomes. Table S3. Missing value imputation details for sample covariates. Fig. S1. Visualisation of the time-varying hazard effects. Table S4. Hazard ratios, 95% confidence intervals and p-values for associations between NHS Health Check and incident diagnoses. Fig. S2. Sensitivity analysis with stratified time periods. Table S5. E-value lower bounds for significant hazard ratios. [file 12916_2023_3187_MOESM1_ESM.docx]

**Additional file 1**

**NHS Health Check attendance is associated with reduced multiorgan disease risk:
a matched cohort study in the UK Biobank**

Celeste McCracken, Zahra Raisi-Estabragh, Liliana Szabo, John Robson, Betty Raman,
Anya Topiwala, Adriana Roca-Fernández, Masud Husain, Steffen E. Petersen,^,^ Stefan Neubauer, and Thomas E. Nichols

[Checklist S1: STROBE 2](#_Toc149816239)

[Table S1: Codes for NHS Health Check exposure 3](#_Toc149816240)

[Table S2: UK Biobank fields and codes for ascertainment of outcomes 4](#_Toc149816241)

[Table S3: Missing value imputation details for sample covariates 7](#_Toc149816242)

[Figure S1: Visualisation of the time-varying hazard effects 8](#_Toc149816243)

[Table S4: Associations between NHS Health Check and incident diagnoses 9](#_Toc149816244)

[Figure S2: Sensitivity analysis with stratified time periods 10](#_Toc149816245)

[Table S5: E-value lower bounds for significant hazard ratios 11](#_Toc149816246)

## Checklist S1: STROBE

STROBE Statement—Checklist of items that should be included in reports of ***case-control studies***

| **Section** | **Item No** | **Recommendation** | **Section/ Exhibit** |
| --- | --- | --- | --- |
| Title and abstract |  |  |  |
|  | 1 | (a) Indicate the study’s design with a commonly used term in the title or the abstract | Title |
|  |  | (b) Provide in the abstract an informative and balanced summary of what was done and what was found | Abstract |
| Introduction |  |  |  |
| Background/ rationale | 2 | Explain the scientific background and rationale for the investigation being reported | Introduction: Paras 1-3 |
| Objectives | 3 | State specific objectives, including any prespecified hypotheses | Introduction: Para 4 |
| Methods |  |  |  |
| Study design | 4 | Present key elements of study design early in the paper | Methods |
| Setting | 5 | Describe the setting, locations, and relevant dates, including periods of recruitment, exposure, follow-up, and data collection | Methods: Paras 2, 5 |
| Participants | 6 | (a) Give the eligibility criteria, and the sources and methods of case ascertainment and control selection. Give the rationale for the choice of cases and controls | Methods: Para 3, Fig 1 |
|  |  | (b) For matched studies, give matching criteria and the number of controls per case | Methods: Para 3, Fig 1 |
| Variables | 7 | Clearly define all outcomes, exposures, predictors, potential confounders, and effect modifiers. Give diagnostic criteria, if applicable | Methods: Para 3-6 |
| Data sources/ measurement | 8* | For each variable of interest, give sources of data and details of methods of assessment (measurement). Describe comparability of assessment methods if there is more than one group | Tables S1-S3 |
| Bias | 9 | Describe any efforts to address potential sources of bias | Methods: Statistical analysis, Limitations |
| Study size | 10 | Explain how the study size was arrived at | Fig1 |
| Quantitative variables | 11 | Explain how quantitative variables were handled in the analyses. If applicable, describe which groupings were chosen and why | Methods: Para 6 |
| Statistical methods | 12 | (a) Describe all statistical methods, including those used to control for confounding | Methods: Statistical analysis |
|  |  | (b) Describe any methods used to examine subgroups and interactions | N/A |
|  |  | (c) Explain how missing data were addressed | Table S3 |
|  |  | (d) If applicable, explain how matching of cases and controls was addressed | Methods: Para 3 |
|  |  | (e) Describe any sensitivity analyses | Methods: Statistical analysis |
| Results |  |  |  |
| Participants | 13* | (a) Report numbers of individuals at each stage of study—eg numbers potentially eligible, examined for eligibility, confirmed eligible, included in the study, completing follow-up, and analysed | Fig 1 |
|  |  | (b) Give reasons for non-participation at each stage | Fig1 |
|  |  | (c) Consider use of a flow diagram | Fig1 |
| Descriptive data | 14* | (a) Give characteristics of study participants (eg demographic, clinical, social) and information on exposures and potential confounders | Table 1 |
|  |  | (b) Indicate number of participants with missing data for each variable of interest | Table S3 |
| Outcome data | 15* | Report numbers in each exposure category, or summary measures of exposure | Table1 |
| Main results | 16 | (a) Give unadjusted estimates and, if applicable, confounder-adjusted estimates and their precision (eg, 95% confidence interval). Make clear which confounders were adjusted for and why they were included | Results: Para 4-6, Table S4 |
|  |  | (b) Report category boundaries when continuous variables were categorized | N/A |
|  |  | (c) If relevant, consider translating estimates of relative risk into absolute risk for a meaningful time period | N/A |
| Other analyses | 17 | Report other analyses done—eg analyses of subgroups and interactions, and sensitivity analyses | 13-14, Fig S2 |
| Discussion |  |  |  |
| Key results | 18 | Summarise key results with reference to study objectives | Discussion: Para 1 |
| Limitations | 19 | Discuss limitations of the study, taking into account sources of potential bias or imprecision. Discuss both direction and magnitude of any potential bias | Limitations |
| Interpretation | 20 | Give a cautious overall interpretation of results considering objectives, limitations, multiplicity of analyses, results from similar studies, and other relevant evidence | Discussion + Conclusion |
| Generalisability | 21 | Discuss the generalisability (external validity) of the study results | Limitations: Para 1 |
| Other information |  |  |  |
| Funding | 22 | Give the source of funding and the role of the funders for the present study and, if applicable, for the original study on which the present article is based | Declarations |

*Give information separately for cases and controls.

**Note:** An Explanation and Elaboration article discusses each checklist item and gives methodological background and published examples of transparent reporting. The STROBE checklist is best used in conjunction with this article (freely available on the Web sites of PLoS Medicine at http://www.plosmedicine.org/, Annals of Internal Medicine at http://www.annals.org/, and Epidemiology at http://www.epidem.com/). Information on the STROBE Initiative is available at http://www.strobe-statement.org.

## Table S1: Codes for NHS Health Check exposure

| Record text | Codes | Records | Participants |
| --- | --- | --- | --- |
| NHS Health Check completed | XaRBQ, 8BAg. | 45,500 | 41,576 |
| NHS Health Check programme | XaR6f, 6B5.. | 6,379 | 6,101 |
| NHS Health Check raising awareness about dementia and memory clinics | XaaD1, 67DF. | 2,438 | 2,380 |
| NHS Health Check annual review | XaXIS, 6AH.. | 1,632 | 1,397 |
| NHS Health Check completed by third party | XaZPq, 8BAg0 | 486 | 479 |
| NHS Health Check follow up | Xab3O, 8HBR. | 26 | 26 |
| NHS Health Check commenced | Xaeab | 3 | 3 |
| Keep well programme | 6B3.., XaNPq | 1,224 | 1,152 |
| Keep Well health check | XaZHe, 8BAn. | 200 | 182 |
| Keep Well health check review | XaZHf, 6AM.. | 72 | 69 |
|  |  | 57,960 | 50,984 |

## Table S2: UK Biobank fields and codes for ascertainment of outcomes

| Source | UK Biobank Field ID or code |
| --- | --- |
| Stroke |  |
| Algorithm | 42006 |
| Self-report (20002) | 1081, 1086, 1491, 1583 |
| ICD10 | I60, I61, I63, I64 |
| ICD9 | 430, 431, 434, 436 |
| Diagnosed by doctor (6150, 6152) | 3, 4056 |
| First occurrences | 131360, 131362, 131366, 131368 |
| Primary care | G60.., G600., G601., G602., G603., G604., G605., G606., G60X., G60z., G61.., G610., G611., G612., G613., G614., G615., G616., G617., G618., G619., G61X., G61X0, G61X1, G61z., G63y0, G63y1, G64.., G640., G6400, G641., G6410, G64z., G64z0, G64z1, G64z2, G64z3, G64z4, G65z1, G66.., G660., G661., G662., G663., G664., G665., G666., G667., G668., G669., G6760, G6W.., G6X.., Gyu60, Gyu61, Gyu62, Gyu63, Gyu64, Gyu6E, Gyu6F, Gyu6G, X00D3, X00D4, X00D5, X00D6, X00D7, X00D8, X00D9, X00DA, X00DD, X00DE, X00DF, X00Df, X00DG, X00Dg, X00DI, X00DJ, X00DK, X00DM, X00DN, X00DO, X00DP, X00DQ, X00DR, X00DS, X00DT, X204F, Xa00I, Xa00J, Xa00K, Xa01b, Xa01c, Xa01h, Xa01i, Xa01j, Xa01k, Xa01l, Xa01m, Xa01o, Xa0Bj, Xa0kZ, Xa0N3, Xa0NJ, Xa0NK, Xa0NN, Xa0NO, Xa0NQ, Xa0NS, Xa0NV, Xa0NW, Xa0NX, Xa1hE, XaB4Z, XaBE2, XaBEC, XaBED, XaJgQ, XE0VJ, XE2aB |
| All-cause dementia |  |
| Algorithm | 42018, 42020, 42022, 42024 |
| Self-report (20002) | 1263 |
| ICD10 | F00, F01, F02, F03, F051, G30, G31 |
| ICD9 | 290, 331 |
| First occurrences | 130836, 130838, 130840, 130842, 131036, 131038 |
| Primary care | .1461, .3AE3, .3AE4, .3AE5, .3AE6, .6AB., .E11., .E111, .E112, .E113, .E114, .E115, .E116, .E11Z, .F21Z, .F371, .G78., 1461, 2233, 38C13, 3AE3., 3AE4., 3AE5., 3AE6., 6AB.., 8BM02, 8BM50, 8BM60, 8BPa., 8CMe0, 8CMG2, 8CMZ., 8CMZ0, 8CMZ1, 8CMZ2, 8CMZ3, 8CSA., 8Hla., 8IAe0, 8IAe2, A411., A4110, E00.., E000., E001., E0010, E0011, E0012, E0013, E001z, E002., E0020, E0021, E002z, E003., E004., E0040, E0041, E0042, E0043, E004z, E012., E02y1, E0300, E0301, E0302, E0303, E0304, E0310, E0311, E0312, E0313, E0314, E041., Eu00., Eu000, Eu001, Eu002, Eu00z, Eu01., Eu010, Eu011, Eu012, Eu013, Eu01y, Eu01z, Eu02., Eu020, Eu021, Eu022, Eu023, Eu024, Eu025, Eu02y, Eu02z, Eu04., Eu040, Eu041, Eu04y, Eu04z, Eu106, Eu107, F1..., F10.., F10y., F10y0, F10y1, F10y2, F10yz, F10z., F11.., F110., F1100, F1101, F111., F112., F116., F118., F11x0, F11x2, F11x7, F11x9, F11xz, F11y., F11y2, F11yz, F11z., F1440, F21y2, Fyu30, Fyu31, Ub1T6, X002m, X002U, X002V, X002W, X002w, X002x, X002y, X002z, X0030, X0031, X0032, X0033, X0034, X0035, X0036, X0037, X0039, X003A, X003B, X003C, X003D, X003E, X003F, X003G, X003H, X003I, X003J, X003l, X003m, X003P, X003R, X003T, X003V, X003W, X003X, X004B, X004E, X005K, X005L, X005M, X005N, X005O, X005P, X00R0, X00R2, X00RI, X00Rk, X77qx, Xa0fZ, Xa0lH, Xa0sC, Xa0sE, Xa1GB, Xa25J, Xa3ez, Xa7nD, XaA1S, XaaBZ, XaaeA, XaaiW, Xabd2, Xabd3, XabEk, XabEl, XabtQ, XabVp, XacIx, XacIy, XacIz, XacJ0, XacLx, Xacly, Xaclz, XacM2, XaE74, Xaefu, XaIKB, XaIKC, XaIRJ, XaJBQ, XaJBU, XaJBV, XaJBW, XaJBX, XaJPy, XaKyY, XaMGF, XaOfZ, XaPws, XaYFR, XaYPX, XaZqJ, XaZWz, XE15F, XE17j, XE1aG, XE1Xr, XE1Xs, XE1Xu, XE1Z6, XE1Z7, XE1Z8 |
| Myocardial infarction |  |
| Algorithm | 42000 |
| Self-report (20002) | 1075 |
| ICD10 | I21, I22, I23, I241, I252 |
| ICD9 | 410, 411, 412, 429 |
| Diagnosed by doctor (6150, 6152) | 1, 3894 |
| First occurrences | 131298, 131300, 131302 |
| Primary care | 14A3., 14A4., 322.., 3222, 889A.00, G30.., G300., G301., G3010, G3011, G301z, G302., G303., G304., G305., G306., G307., G3070, G3071, G308., G309., G30A., G30B., G30X., G30X0, G30y., G30y0, G30y1, G30y2, G30yz, G30z., G31.., G310., G3110, G3115, G312., G31y., G31y0, G31y1, G31y2, G31y3, G31yz, G32.., G34.., G340., G3400, G3401, G341., G3410, G3411, G3412, G3413, G341z, G342., G343., G344., G34y., G34y0, G34y1, G34yz, G34z., G34z0, G35.., G350., G351., G353., G35X., G36.., G360., G361., G362., G363., G364., G365., G366., G38.., G380., G381., G382., G383., G384., G38z., G3y.., G3z.., G501., G501.00, G5y2., G704., G72B1, Gyu31, Gyu32, Gyu33, Gyu34, Gyu35, Gyu36, X2006, X200a, X200d, X200D, X200e, X200G, X200H, X200I, X200J, X200K, X200L, X200M, X200N, X200O, X200P, X200Q, X200R, X200S, X200T, X200U, X200V, X200W, X200x, X200X, X200Y, X200Z, X201u, X2021, X202q, X202r, X203e, X204f, X204q, X77vP, X782C, X782F, Xa0YL, Xa6Yx, XaAC3, XaAzi, XaBL1, XaEgZ, XaeVd, XaFsH, XaG1Q, XaIf1, XaINF, XaIwM, XaIwY, XaJX0, XC0bX, XE0Uk, XE0WG, XE2aA, XM0rN, XM1Qk, XSDT6 |
| Atrial fibrillation |  |
| Self-report (20002) | 1471 |
| ICD10 | I48, I480, I481, I482, I489 |
| ICD9 | 4273 |
| First occurrences | 131350 |
| Primary care | .14AN, .662S, .6A9., .8666, .G67., .G670, 14AN., 662S., 6A9.., 7936A, G573., G5730, G5731, G5732, G5733, G5734, G5735, G5736, G5737, G5738, G5739, G573z, X202R, X202S, Xa2E8, Xa3rp, Xa7nI, XaaUH, XaDv6, XaEga, XaeUP, XaeUQ, XaeUR, Xafis, XaIIT, XaMGD, XaOfa, XaOft, XE0Wk, Y7820 |
| Heart failure |  |
| Self-report (20002) | 1076 |
| ICD10 | I500, I501, I509 |
| ICD9 | 4280, 4281, 4289 |
| First occurrences | 131354 |
| Primary care | .14A6, .14AM, .1O1., .662p, .662T, .662W, .8H2S, .8HBE, .G6A., .G6A1, .G6A2, .G6AZ, 14A6., 14AM., 1O1.., 662p., 662T., 662W., 8H2S., 8HBE., G1yz1, G232., G234., G58.., G580., G5800, G5801, G5802, G5803, G5804, G581., G5810, G582., G583., G584., G58z., G5y4z, SP111, X102Y, X202k, X202l, XaBwi, XaEgY, XafeB, XaIIU, XaIpn, XaIQM, XaIQN, XaKNW, XaLon, XaO5n, XaWyi, XaZIC, XE0V8, XE0V9, XE0Wo, XE2QG, Y27da, Y2b96 |
| Acute kidney injury |  |
| Self-report (20002) | 1192 |
| ICD10 | N17 |
| ICD9 | 584 |
| First occurrences | 132030 |
| Primary care | .J15., 14D8., 451L., K034., K035., K04.., K040., K041., K042., K043., K0430, K0431, K0432, K0433, K0434, K044., K045., K046., K0460, K0461, K047., K048., K049., K04A., K04B., K04C., K04D., K04E., K04y., K04z., K13D., Kyu20, L0931, L393., L3930, L3931, L3932, L393z, SK08., X30Im, X30Ir, X30Is, X30Iu, X30J5, X30J6, Xa6nr, Xa85t, Xaa8O, Xaa8P, Xaa8Q, XabmE, XaebJ, XaPwv, XaZ6J, XaZe5, XaZPp, XaZPs, XaZPt, XaZPu, XaZSx, XaZUa, XaZUY, XaZUZ, XaZYv, XaZZ0, XaZZ2, XE1oB, XE2QM |
| Chronic kidney disease |  |
| Algorithm | 42026 |
| Self-report (20002) | 1193 |
| Self-report (20004) | 1195, 1580, 1581, 1582 |
| ICD10 | N165, N180, N183, N184, N185, N188, N189, T824, T861, Y602, Y612, Y622, Y841, Z490, Z491, Z492, Z940, Z992 |
| ICD9 | 585, 5859 |
| Primary care | .1Z12, .1Z13, .1Z14, .1Z15, .1Z16, .1Z1B, .1Z1C, .1Z1D, .1Z1E, .1Z1F, .1Z1G, .1Z1H, .1Z1J, .1Z1K, .1Z1L, 1Z12., 1Z13., 1Z14., 1Z15., 1Z16., 1Z1a., 1Z1b., 1Z1B., 1Z1c., 1Z1C., 1Z1d., 1Z1D., 1Z1e., 1Z1E., 1Z1f., 1Z1F., 1Z1G., 1Z1H., 1Z1J., 1Z1K., 1Z1L., 1Z1T., 1Z1V., 1Z1W., 1Z1X., 1Z1Y., 1Z1Z., K05.., K050., K053., K054., K055., K0D.., X30In, X30J0, X30J1, XacAb, XacAd, XacAe, XacAf, XacAh, XacAi, XacAM, XacAN, XacAO, XacAV, XacAW, XacAX, XaLHI, XaLHJ, XaLHK, XaNbn, XaNbo, XaO3t, XaO3u, XaO3v, XaO3w, XaO3x, XaO3y, XaO3z, XaO40, XaO41, XaO42, XE0df |
| OPCS4 | L741, L742, L743, L744, L745, L746, L748, L749, M012, M013, M014, M015, M018, M019, M023, M084, M172, M174, M178, M179, X401, X402, X403, X404, X405, X406, X407, X408, X409, X411, X412, X418, X419, X421, X428, X429, X431 |
| NAFLD |  |
| ICD10 | K758, K760 |
| Primary care | EMISR4QFA1, J61y1, J61y7, J61y8, XaQIT, J61y9, X307v |
| Alcoholic liver disease |  |
| ICD10 | K70 |
| ICD9 | 5710, 5711, 5712, 5713 |
| First occurrences | 131658 |
| Primary care | .I72., .I722, .I723, G8523, J610., J611., J612., J6120, J613., J6130, J617., J6170, X306r, X3071, X3072, X3073, XaBE3, XaC1d, XE0b4, XE0dD, XE0dF |
| Liver cirrhosis |  |
| Self-report (20002) | 1141, 1158 |
| ICD10 | I850, I859, I864, I982, I983, K740, K741, K742, K746, K766, K767 |
| ICD9 | 4560, 4561, 5715, 5722, 5723, 5724 |
| First occurrences | 131666 |
| Primary care | .I725, 2485, G85.., G850., G851., G852., G8520, G8521, G8522, G852z, G857., G858., Gyu94, HNG0090, J61.., J615., J6150, J6151, J6152, J6153, J6154, J6155, J6156, J6157, J6158, J6159, J615A, J615B, J615C, J615D, J615E, J615F, J615G, J615H, J615y, J615z, J616., J6160, J6161, J6162, J616z, J61y., J61y3, J61y4, J61y5, J61y6, J6356, Jyu71, X307L, X307M, X307N, X307O, X307P, X307S, X307T, X307U, X307V, Xa9C7, XaBM6, XE0b5, XE0b7, XE0bA, XE2up |
| Liver failure |  |
| Self-report (20002) | 1158 |
| ICD10 | K72, T864, Z944 |
| ICD9 | 570 |
| First occurrences | 131662 |
| Primary care | .79L8, .I71., 7800, 78000, 78001, 7L1f., 7L1fy, 7L1fz, J60.., J600., J6000, J6001, J6002, J600z, J601., J6010, J6011, J6012, J601z, J60z., J6130, J61y0, J622., J625., J62y., SP086, SP142, X0042, X0058, X20a8, X3073, X3076, X3077, X3078, X3079, X307A, X307C, X307x, X307z, Xa8Df, XaMuG, XaMuH, XaMuI, XE0dB, ZV427 |
| OPCS4 | J01, X43 |
| Diabetes |  |
| Self-report (20002) | 1220, 1222, 1223 |
| ICD10 | E10, E11, E13, E14, G590, G632, H280, H360, M142, N083, O240, O241, O243, O244, O249, Y423 |
| Diagnosed by doctor | 2443, 2976, 2986 |
| Medications (20003) | 1140883066 |
| Medications (6153, 6177) | 3 |
| First occurrences | 130706, 130708, 130712, 130714 |
| Primary care | C1000, C1001, C100z, C101., C1010, C1011, C101y, C101z, C102., C1020, C1021, C102z, C103., C1030, C1031, C103y, C103z, C104y, C105., C1050, C1051, C105y, C105z, C106., C1060, C1061, C106y, C106z, C107., C1070, C1071, C1072, C1073, C1074, C107y, C107z, C108., C1080, C1081, C1082, C1083, C1084, C1085, C1086, C1088, C1089, C108A, C108E, C108y, C108z, C109., C1090, C1091, C1092, C1093, C1094, C1095, C1097, C1099, C109D, C109J, C109K, C10B., C10B0, C10C., C10D., C10E., C10E0, C10E1, C10E2, C10E3, C10E4, C10E5, C10E6, C10E8, C10E9, C10EA, C10EE, C10EM, C10EN, C10ER, C10F., C10F0, C10F1, C10F2, C10F3, C10F4, C10F5, C10F7, C10F8, C10F9, C10FD, C10FJ, C10FK, C10FN, C10FP, C10FS, C10G., C10G0, C10H., C10H0, C10J., C10J0, C10K., C10K0, C10L., C10L0, C10M., C10M0, C10N., C10N0, C10N1, C10P0, C10P1, C10Q., C10y., C10y0, C10y1, C10yy, C10yz, C10z., C10z0, C10z1, C10zy, C10zz, C11y0, Cyu20, Cyu23, F374., F374z, F4205, F464., F4643, F464z, Fyu6D, Fyu73, Fyu74, Fyu75, Fyu76, Fyu77, Fyu78, Fyu79, FyuE3, FyuE4, FyuE5, FyuF9, K00y0, K01x., K01xz, K02y0, K03y0, K0A6., K0A7., Kyu00, Kyu01, Kyu02, Kyu03, Kyu04, Kyu05, Kyu06, L180., L1800, L1801, L1802, L1803, L1804, L1805, L1806, L1807, L1808, L1809, L180A, L180B, L180X, L180z, Lyu29, M0372, M2710, M2711, M2712, N03.., N030., N0302, N031., N03x., N03x0, N03x1, N03x2, N03x3, N03x4, N03x5, N03x6, N03x7, N03x8, N03x9, N03xA, N03xB, N03xC, N03xD, N03xE, N03xF, N03xG, N03xH, N03xJ, N03xK, N03y., N03z., Nyu1C, Nyu1D, Nyu1E, Nyu1F, R0542, R0543, X008t, X00cE, X40JI, X40Jj, X40Jk, X40KH, Xaagd, XacoB, XaE69, XaE6A, XaJlL, XaJlN, XaJlO, XaJlR, XaMzI, .66An, .C2C., .C2C0, 66An., C1087, C108B, C108C, C108D, C108F, C108G, C108H, C108J, C10E7, C10EB, C10EC, C10ED, C10EF, C10EG, C10EH, C10EJ, C10EK, C10EL, C10EP, C10EQ, X40J4, Xa2h9, Xa4g7, Xaage, XaELP, XaEnn, XaEno, XaF04, XafjT, XaFm8, XaFmK, XaFmL, XaFmM, XaFWG, XaIzM, XaIzN, XaJSr, XaKyW, XaMhJ, XaX49, XaXZv, XM19i, .66Ao, .C2A., .C2D., .C2D0, 66Ao., C1096, C109A, C109B, C109C, C109E, C109F, C109G, C109H, C10F6, C10FA, C10FB, C10FC, C10FE, C10FF, C10FG, C10FH, C10FL, C10FM, C10FQ, C10FR, X40J5, X40J6, X40JJ, Xa2hA, XaELQ, XaEnp, XaEnq, XaF05, XaFmA, XaFn7, XaFn8, XaFn9, XaFWI, XaIfG, XaIfI, XaIrf, XaIzQ, XaIzR, XaJQp, XaKyX, XaMhK, XaXZR, XM19j, .66A5, .66Am, .66Ap, .66AV, .C22., 66Am., 66Ap., 66AV., 66Aw., 66o6., 7L100, 7L198, EMISNQCO166, EMISNQIN19, EMISNQIN49, EMISNQIN92, EMISNQSE45, f111., f12.., f121., f12E., f12W., f12X., f12y., f12z., f13.., f131., f133., f136., f13A., f14.., f14w., f14x., f14y., f14z., f15.., f15x., f15y., f15z., f2..., f21.., f21z., f22.., f221., f22y., f22z., f23.., f23z., f24.., f24z., f25.., f251., f25s., f25W., f25X., f25Y., f25Z., f26.., f26z., f27.., f28.., f29.., f291., f292., f293., f29A., f2A.., f2Ax., f2Ay., f2Az., f2B.., f2B2., f2B4., f2B6., f2C.., f2C2., fw1.., fw2.., ph2.., ph34., ph35., ph36., ph3c., ph3J., ph3K., ph3N., ph3P., ph3Q., ph3T., ph3U., ph3V., X71b8, X75XP, Xa5vt, Xa5vu, Xa5XQ, Xa5XR, XaMdq, XaP63, XaR7E, XaXfW, Y1206 |
| Hypertension |  |
| Self-report (20002) | 1065, 1072 |
| ICD10 | I10 |
| Diagnosed by doctor (6150, 6152) | 2966, 4 |
| Medications (6153, 6177) | 2 |
| First occurrences | 131286 |
| Primary care | .14A2, .6627, .6628, .662d, .662F, .662O, .662P, .G3.., .G31., .G35., .G36., 14A2., 6627, 6628, 662d., 662F., 662O., 662P., 662P0, 7Q01., 7Q01z, EMISHGT69, EMISNQST25, G2..., G20.., G200., G201., G202., G203., G20z., G24.., G240., G24z., G24zz, G25.., G250., G251., G26.., G27.., G28.., G2y.., G2z.., HNGZ016, Xa3fQ, Xa8HD, XaIyE, XaM5f, XaM5p, XaXOi, XaZbz, XaZWn, XE0Ub, XE0Uc, XE0Ud, XE0W8, XM1YA, XSDSb |
| High cholesterol |  |
| Self-report (20002) | 1473 |
| ICD10 | E780, E782, E783, E784, E785 |
| Medications (6153, 6177) | 1 |
| First occurrences | 130814 |
| Primary care | .C521, C32.., C320., C3200, C3201, C3202, C3203, C3204, C3205, C3206, C320y, C320z, C321., C3210, C322., C3220, C323., C324., C325., C3250, C3251, C3252, C3253, C325z, C328., C329., C32y., C32yz, C32z., Cyu8D, Cyu8E, X003s, X00dK, X20DV, X20DW, X40Vd, X40Vf, X40Vg, X40Vh, X40VH, X40Vi, X40VI, X40Vm, X40Vn, X40Vo, X40Vp, X40Vq, X40Vs, X40Vt, X40VT, X40VW, X40VY, X40Wx, X40Wy, X40Wz, X40X0, X40X1, X40X2, X40X3, X40X5, X40X6, X40X7, X40X9, X40XA, X40XH, X40XI, X40XO, X40XP, X50DQ, X50Fh, X50Fi, X50Fm, X50Fp, X76tz, X78qa, Xa9As, Xa9At, XaL5p, XaR4h, XaR4i, XaR4k, XaRG5, XE11R, XE11T, XE11U, XE11V, XE11W, XE13C |

**Table S2 footnote**: ICD10 codes are drawn from fields 41270, 41280, 41234 and 41259; ICD9 codes are drawn from fields 41271, 41281, 41234 and 41259; OPCS4 codes are drawn from fields 41272, 41282, 41149 and 41259; Primary care codes are drawn from field 42040. Death codes are drawn from fields 40000, 40001 and 40023. Where a 3-digit code is given, this includes all 4-digit sub-codes, for example, I46 includes I462, I468 and I469.

## Table S3: Missing value imputation details for sample covariates

| Covariate | Missing count | Missing % |
| --- | --- | --- |
| Body mass index | 677 | 0.50% |
| Waist-hip ratio | 493 | 0.36% |
| Systolic blood pressure | 295 | 0.22% |
| Summed MET-minutes per week | 770 | 0.56% |
| Fresh vegetable/salad daily intake | 1,807 | 1.32% |
| Alcohol intake frequency | 298 | 0.22% |
| Educational attainment | 1,057 | 0.77% |
| Townsend deprivation score | 191 | 0.14% |

**Table S3 footnote:** Missing values in covariate variables in the main data set (n= 140,899). Missing values were imputed together using multiple imputation with chained equations (MICE) using the mice package in R, using 20 iterations to create a single imputed replicate. No imputation was used for exposure or outcomes.

## Figure S1: Visualisation of the time-varying hazard effects


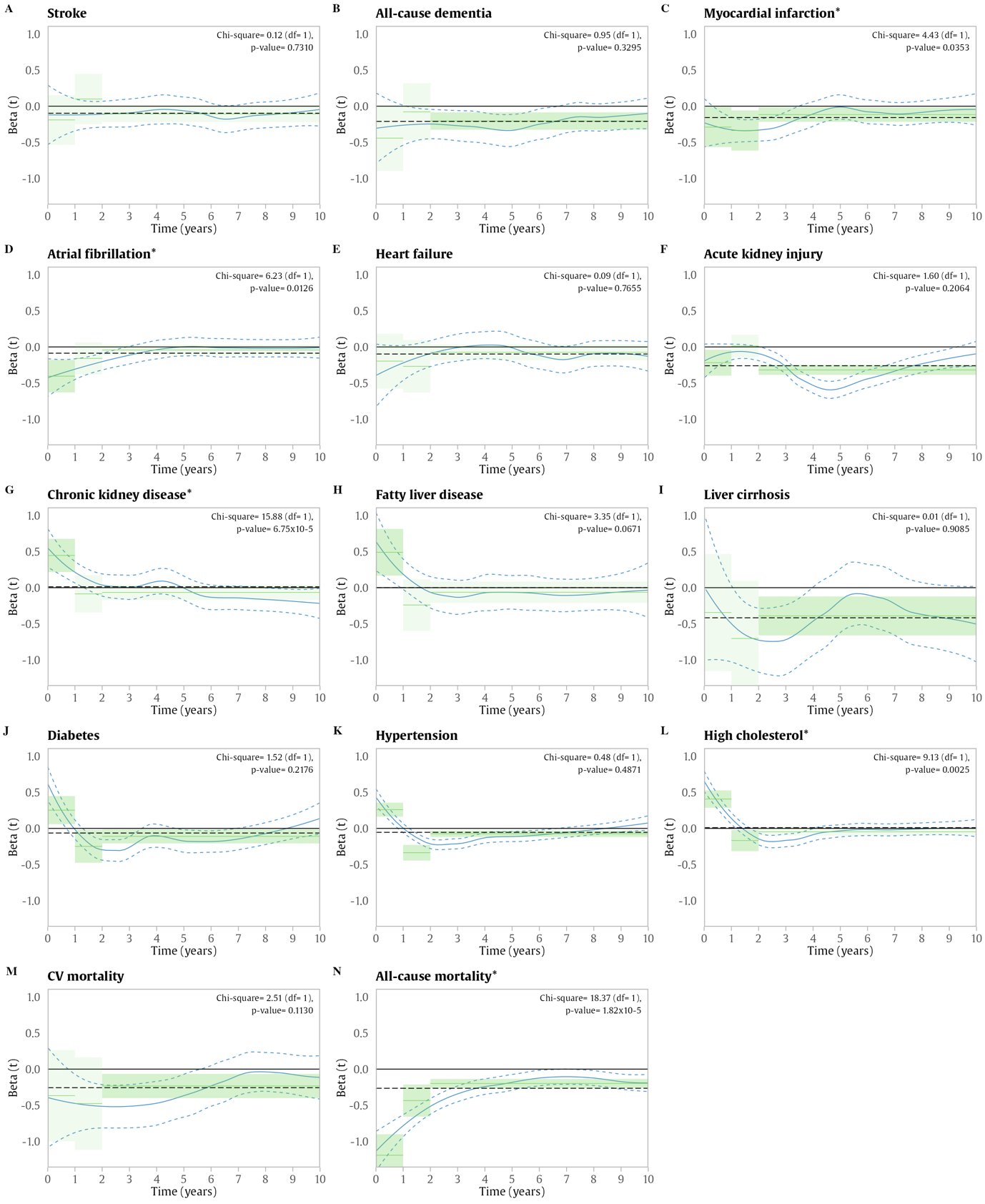


**Figure S1 footnote:** Each panel shows the smoothed curve for Schoenfeld residuals (in blue), for the treatment variable (NHS Health Check) from aligned-start Cox regression models. Where the heading of the panel is marked with an asterisk (*), the test for proportional hazards has failed (p-value < 0.05). The zero line is shown in solid black with the non-stratified treatment effect in dashed black line. Green rectangles indicate the 95% confidence regions for the treatment coefficient in each time strata (the first year, the second year, and time after the 2-year mark). Where the stratified effect is non-significant, this is shown in lighter transparent green.

## Table S4: Associations between NHS Health Check and incident diagnoses

|  | Ignoring the first (months) | Time-varying Cox regression | | Aligned-start Cox regression | |
| --- | --- | --- | --- | --- | --- |
| Outcome |  | HR (95% CI) | p-value | HR (95% CI) | p-value |
| Hypertension | 0 | 1.06* [1.02, 1.10] | 0.0031 | 0.95* [0.91, 0.98] | 0.0043 |
|  | 12 | 0.84* [0.81, 0.88] | 1.15x10-17 | 0.90* [0.86, 0.93] | 1.80x10-7 |
|  | 24 | 0.73* [0.70, 0.76] | 4.95x10-57 | 0.93* [0.89, 0.97] | 0.0016 |
| Diabetes | 0 | 1.05 [0.96, 1.14] | 0.2773 | 0.94 [0.86, 1.02] | 0.1227 |
|  | 12 | 0.82* [0.75, 0.89] | 3.80x10-6 | 0.88* [0.80, 0.96] | 0.0041 |
|  | 24 | 0.69* [0.63, 0.75] | 1.99x10-17 | 0.90 [0.82, 0.99] | 0.0376 |
| High cholesterol | 0 | 1.11* [1.06, 1.16] | 2.49x10-5 | 1.01 [0.96, 1.06] | 0.6673 |
|  | 12 | 0.88* [0.84, 0.93] | 2.08x10-7 | 0.94* [0.89, 0.99] | 0.0153 |
|  | 24 | 0.77* [0.73, 0.81] | 5.96x10-27 | 0.95 [0.90, 1.01] | 0.0813 |
| Stroke | 0 | 0.97 [0.88, 1.07] | 0.5722 | 0.91 [0.82, 1.00] | 0.0622 |
|  | 12 | 0.88* [0.80, 0.97] | 0.0142 | 0.92 [0.82, 1.02] | 0.1124 |
|  | 24 | 0.78* [0.70, 0.86] | 1.70x10-6 | 0.90 [0.81, 1.01] | 0.071 |
| All-cause dementia | 0 | 0.85* [0.76, 0.95] | 0.0053 | 0.81* [0.72, 0.91] | 2.23x10-4 |
|  | 12 | 0.80* [0.72, 0.90] | 1.01x10-4 | 0.82* [0.73, 0.92] | 9.57x10-4 |
|  | 24 | 0.72* [0.64, 0.81] | 1.87x10-8 | 0.81* [0.72, 0.92] | 7.88x10-4 |
| Myocardial infarction | 0 | 0.95 [0.87, 1.04] | 0.2957 | 0.85* [0.78, 0.93] | 5.21x10-4 |
|  | 12 | 0.84* [0.77, 0.92] | 1.11x10-4 | 0.87* [0.79, 0.95] | 0.0031 |
|  | 24 | 0.74* [0.67, 0.81] | 6.67x10-11 | 0.89 [0.81, 0.98] | 0.0233 |
| Atrial fibrillation | 0 | 0.98 [0.91, 1.04] | 0.4849 | 0.92* [0.86, 0.98] | 0.0116 |
|  | 12 | 0.89* [0.83, 0.95] | 4.88x10-4 | 0.95 [0.88, 1.02] | 0.1388 |
|  | 24 | 0.79* [0.74, 0.85] | 1.12x10-11 | 0.96 [0.89, 1.03] | 0.2775 |
| Heart failure | 0 | 0.97 [0.88, 1.07] | 0.5409 | 0.91 [0.82, 1.00] | 0.0561 |
|  | 12 | 0.89* [0.81, 0.99] | 0.0279 | 0.91 [0.82, 1.01] | 0.0911 |
|  | 24 | 0.82* [0.74, 0.91] | 1.22x10-4 | 0.93 [0.84, 1.04] | 0.2048 |
| Acute kidney injury | 0 | 0.84* [0.79, 0.89] | 9.02x10-9 | 0.77* [0.73, 0.82] | 8.54x10-18 |
|  | 12 | 0.75* [0.71, 0.80] | 4.02x10-20 | 0.76* [0.72, 0.81] | 5.56x10-17 |
|  | 24 | 0.61* [0.57, 0.65] | 1.45x10-51 | 0.72* [0.68, 0.77] | 2.63x10-20 |
| Chronic kidney disease | 0 | 1.15* [1.05, 1.26] | 0.0025 | 1.01 [0.93, 1.11] | 0.7934 |
|  | 12 | 0.91 [0.83, 1.00] | 0.0400 | 0.94 [0.85, 1.03] | 0.1944 |
|  | 24 | 0.76* [0.69, 0.84] | 1.15x10-8 | 0.94 [0.85, 1.05] | 0.256 |
| Fatty liver disease | 0 | 1.17* [1.03, 1.33] | 0.0161 | 1.00 [0.88, 1.13] | 0.9961 |
|  | 12 | 0.91 [0.80, 1.04] | 0.1540 | 0.92 [0.80, 1.05] | 0.2062 |
|  | 24 | 0.76* [0.66, 0.86] | 3.66x10-5 | 0.94 [0.81, 1.09] | 0.4294 |
| Alcoholic liver disease | 0 | 0.85 [0.60, 1.18] | 0.3243 | 0.72 [0.52, 0.98] | 0.0391 |
|  | 12 | 0.73 [0.52, 1.01] | 0.0608 | 0.68* [0.49, 0.95] | 0.0239 |
|  | 24 | 0.58* [0.42, 0.82] | 0.0020 | 0.67* [0.46, 0.95] | 0.0265 |
| Liver cirrhosis | 0 | 0.70* [0.55, 0.90] | 0.0046 | 0.66* [0.52, 0.84] | 7.94x10-4 |
|  | 12 | 0.62* [0.49, 0.80] | 1.71x10-4 | 0.66* [0.51, 0.85] | 0.0012 |
|  | 24 | 0.56* [0.43, 0.72] | 6.19x10-6 | 0.68* [0.52, 0.89] | 0.0047 |
| Liver failure | 0 | 0.81 [0.54, 1.22] | 0.3241 | 0.71 [0.48, 1.06] | 0.0923 |
|  | 12 | 0.70 [0.46, 1.06] | 0.0952 | 0.71 [0.47, 1.09] | 0.1158 |
|  | 24 | 0.54* [0.35, 0.83] | 0.0051 | 0.63 [0.40, 0.99] | 0.0469 |
| CV mortality | 0 | 0.86 [0.73, 1.01] | 0.0643 | 0.77* [0.66, 0.90] | 0.0013 |
|  | 12 | 0.80* [0.68, 0.94] | 0.0072 | 0.78* [0.66, 0.92] | 0.0026 |
|  | 24 | 0.75* [0.64, 0.88] | 4.79x10-4 | 0.79* [0.67, 0.94] | 0.007 |
| All-cause mortality | 0 | 0.86* [0.81, 0.92] | 1.16x10-6 | 0.77* [0.72, 0.81] | 8.31x10-19 |
|  | 12 | 0.83* [0.78, 0.88] | 1.48x10-9 | 0.81* [0.76, 0.86] | 2.36x10-12 |
|  | 24 | 0.77* [0.72, 0.82] | 1.90x10-17 | 0.82* [0.77, 0.87] | 7.21x10-10 |

**Table S4 footnote:** Entries are hazard ratios, 95% confidence intervals and p-values from Cox proportional hazards models associating completed NHS Health Check with new diagnoses received during follow-up. An asterisk (*) indicates p-value significance after applying multiple testing corrections with a 5% false discovery rate. Columns show models that were computed with three outcome exclusion settings (including all outcomes, excluding outcomes in the first 12 months after NHS Health Check, and excluding outcomes in the first 24 months after NHS Health Check). Models are adjusted by age, sex, geographical region, Townsend deprivation score, ethnicity, body mass index, waist-hip ratio, smoking, systolic blood pressure, alcohol intake frequency, physical activity, daily vegetable/salad intake and Charlson comorbidity index.

## Figure S2: Sensitivity analysis with stratified time periods


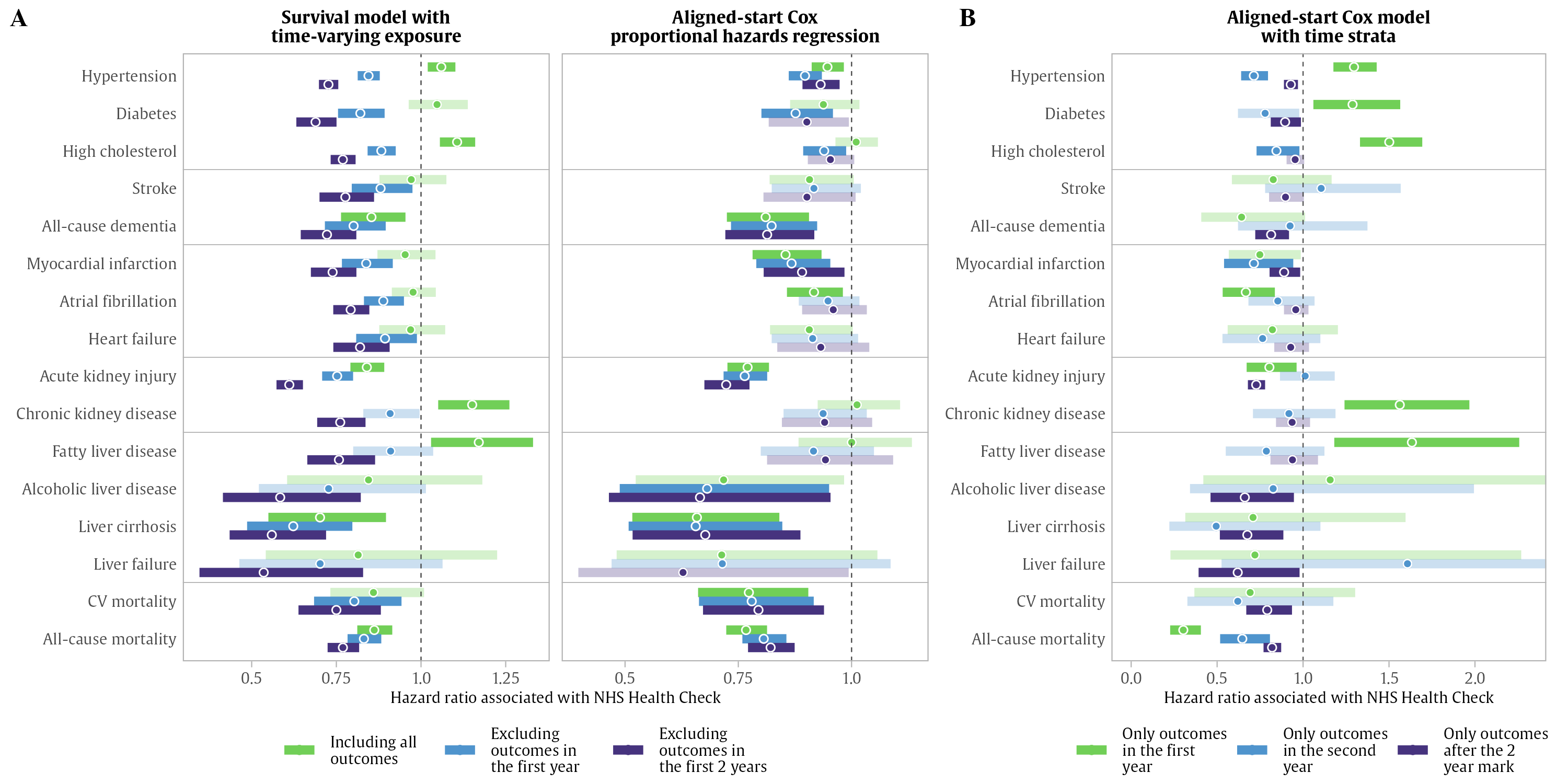


**Figure S2 footnote:** Panel A is a copy of main Figure 5. A new panel (B) showing the results from the matched-sample aligned-start Cox model, with stratified time periods. In contrast to Panel A, Panel B divides follow-up time into three parts; the first year (green) the second year (blue) and follow-up time after the 2-year mark (purple). This is another way to deal with hazards that are not necessarily steady across time.

## Table S5: E-value lower bounds for significant hazard ratios

Translated into their nearest “years of ageing” equivalent based on the outcome-specific
age coefficient in each model.

|  |  | Time-varying Cox regression | | Aligned-start Cox regression | | Heuristic evaluation of longer-term effect? |
| --- | --- | --- | --- | --- | --- | --- |
| Outcome | Ignoring the first k months | HR | Years equivalent  (E-value lower bound) | HR | Years equivalent  (E-value lower bound) |  |
| Hypertension | 0 | 1.06* | 5 years (1.16) | 0.95* | 4 years (1.15) | Strong |
|  | 12 | 0.84* | 10 years (1.54) | 0.90* | 8 years (1.34) | 7.4 |
|  | 24 | 0.73* | 10 years (1.98) | 0.93* | 5 years (1.19) |  |
| Diabetes | 0 | 1.05 |  | 0.94 |  | Moderate |
|  | 12 | 0.82* | 10 years (1.49) | 0.88* | 6 years (1.25) | 5.2 |
|  | 24 | 0.69* | 10 years (2.00) | 0.9 |  |  |
| High | 0 | 1.11* | 4 years (1.30) | 1.01 |  | Moderate |
| cholesterol | 12 | 0.88* | 5 years (1.38) | 0.94* | 2 years (1.12) | 3.2 |
|  | 24 | 0.77* | 9 years (1.78) | 0.95 |  |  |
| Stroke | 0 | 0.97 |  | 0.91 |  | Weak |
|  | 12 | 0.88* | 2 years (1.19) | 0.92 |  | 1.4 |
|  | 24 | 0.78* | 5 years (1.59) | 0.9 |  |  |
| All-cause | 0 | 0.85* | 1 years (1.27) | 0.81* | 2 years (1.44) | Moderate-weak |
| dementia | 12 | 0.80* | 2 years (1.48) | 0.82* | 2 years (1.38) | 2.2 |
|  | 24 | 0.72* | 3 years (1.78) | 0.81* | 2 years (1.40) |  |
| Myocardial | 0 | 0.95 |  | 0.85* | 5 years (1.35) | Moderate |
| infarction | 12 | 0.84* | 6 years (1.41) | 0.87* | 5 years (1.28) | 5.6 |
|  | 24 | 0.74* | 9 years (1.78) | 0.89* | 3 years (1.14) |  |
| Atrial | 0 | 0.98 |  | 0.92* | 1 years (1.16) | Weak |
| fibrillation | 12 | 0.89* | 2 years (1.29) | 0.95 |  | 1.6 |
|  | 24 | 0.79* | 5 years (1.64) | 0.96 |  |  |
| Heart failure | 0 | 0.97 |  | 0.91 |  | Very weak |
|  | 12 | 0.89* | 1 years (1.12) | 0.91 |  | 0.80 |
|  | 24 | 0.82* | 3 years (1.44) | 0.93 |  |  |
| Acute kidney | 0 | 0.84* | 7 years (1.49) | 0.77* | 10 years (1.75) | Strong |
| injury | 12 | 0.75* | 10 years (1.81) | 0.76* | 10 years (1.76) | 10.00 |
|  | 24 | 0.61* | 10 years (2.44) | 0.72* | 10 years (1.90) |  |
| Chronic kidney | 0 | 1.15* | 2 years (1.28) | 1.01 |  | Very weak |
| disease | 12 | 0.91 |  | 0.94 |  | 1.00 |
|  | 24 | 0.76* | 5 years (1.68) | 0.94 |  |  |
| Fatty liver | 0 | 1.17* | 10 years (1.20) | 1 |  | Weak |
| disease | 12 | 0.91 |  | 0.92 |  | 2.00 |
|  | 24 | 0.76* | 10 years (1.58) | 0.94 |  |  |
| Alcoholic liver | 0 | 0.85 |  | 0.72 |  | Moderate |
| disease | 12 | 0.73 |  | 0.68* | 8 years (1.29) | 5.2 |
|  | 24 | 0.58* | 10 years (1.73) | 0.67* | 8 years (1.27) |  |
| Liver cirrhosis | 0 | 0.70* | 10 years (1.47) | 0.66* | 10 years (1.66) | Strong |
|  | 12 | 0.62* | 10 years (1.82) | 0.66* | 10 years (1.64) | 10.00 |
|  | 24 | 0.56* | 10 years (2.13) | 0.68* | 10 years (1.51) |  |
| Liver failure | 0 | 0.81 |  | 0.71 |  | Weak |
|  | 12 | 0.70 |  | 0.71 |  | 2.00 |
|  | 24 | 0.54* | 10 years (1.71) | 0.63 |  |  |
| CV mortality | 0 | 0.86 |  | 0.77* | 3 years (1.45) | Moderate-weak |
|  | 12 | 0.80* | 2 years (1.32) | 0.78* | 3 years (1.41) | 2.8 |
|  | 24 | 0.75* | 4 years (1.53) | 0.79* | 2 years (1.33) |  |
| All-cause | 0 | 0.86* | 3 years (1.41) | 0.77* | 6 years (1.76) | Moderate |
| mortality | 12 | 0.83* | 4 years (1.52) | 0.81* | 5 years (1.61) | 4.25 |
|  | 24 | 0.77* | 5 years (1.75) | 0.82* | 4 years (1.55) |  |

**Table S5 footnote**: E-values measure the strength of the association that an unmeasured confounder would need to have with the outcome in order to nullify the observed hazard ratio. In the interest of space, here we have only reported the e-value lower 95% bound for significant hazard ratios, indicating the strength an unmeasured confounder would need to affect the significance of the measured effect, not necessarily to nullify it. Then we have translated the e-value result into the equivalent effect expressed in terms of years of aging calculated from the outcome-specific age coefficient from each model. For example, in the time-varying model for hypertension, with a latency period of zero, an unmeasured confounder with the equivalent strength of 7 years of aging would render our observed hypertension effect non-significant. On the other hand, in our aligned-start model for heart failure, even a weak unmeasured confounder – equivalent to only two years of aging – could potentially render our observed hazard ratio non-significant. The heuristic evaluation is provided for overview only, and is calculated from the average e-value years (not including time-varying model with k=0), where non-significant effects count as zero, where < 1 = Very weak, 1.1-2.0 = Weak, 2.1-2.9= Moderate-weak, 3.0-6.9 = Moderate, ≥7.0 = strong, i.e., a strong unmeasured confounding factor would be required to render our observed effect non-significant.
